# Efficacy Analysis and Prognostic Impact of Sivelestat Sodium in Coronavirus Disease 2019-Related Acute Respiratory Distress Syndrome

**DOI:** 10.3390/ph17030368

**Published:** 2024-03-12

**Authors:** Xiao Che, Wei Hu, Ziying Zhang, Lexiao Wang, Zhe Xu, Fusheng Wang

**Affiliations:** Senior Department of Infectious Disease, The Fifth Medical Center of PLA General Hospital, Beijing 100039, China

**Keywords:** sivelestat sodium, mortality rate, COVID-19, ARDS

## Abstract

Purpose: This study aimed to evaluate the efficacy of sivelestat sodium on mortality, oxygenation index, and serum markers in patients with acute respiratory distress syndrome (ARDS) associated with Coronavirus Disease 2019 (COVID-19). Methods: A retrospective analysis was conducted on adult inpatients admitted to the Intensive Care Unit (ICU). The study compared clinical characteristics, laboratory indices, and mortality rates between patients treated with and without sivelestat sodium. Cox regression analysis was employed to assess the effect of sivelestat sodium on the risk of death, oxygenation index, and improvement of serum markers in patients with COVID-19-associated ARDS. Results: A total of 110 patients with COVID-19-associated ARDS were included, with 45 patients in the sivelestat group and 65 patients in the control group. The overall patient mortality rate was 69.1%, with 62.2% in the sivelestat group and 73.8% in the control group. After five days of treatment, the median change from baseline in the oxygenation index was 21 mmHg in the medicated group and −31 mmHg in the control group (*p* < 0.05). Analysis of the oxygenation index as a clinical endpoint event showed a significantly higher rate of improvement in the sivelestat group compared to the control group (57.8% vs. 38.5%, *p* < 0.05), and the odds of raising the oxygenation index after treatment were 2.05 times higher in the sivelestat group than in the control group (HR = 2.05, 95%CI: 1.02–4.15, *p* < 0.05). Among patients with a baseline oxygenation index < 200 mmHg, patients in the sivelestat group had an 86% lower risk of death compared to the control group (HR = 0.14, 95%CI: 0.02–0.81, *p* < 0.05). Conclusions: Sivelestat sodium demonstrated a significant improvement in the oxygenation index of patients with COVID-19-associated ARDS and was found to considerably reduce the risk of death in patients with a baseline oxygenation index of <200 mmHg.

## 1. Introduction

Coronavirus Disease 2019 (COVID-19) is a multisystemic disease, with the lungs being the most commonly affected organ [1,2,3]. As the disease progresses, patients can develop viral pneumonia or even acute respiratory distress syndrome (ARDS) [4,5]. ARDS is a severe respiratory dysfunction characterized by hypoxemia, diffuse lung infiltrates, and non-cardiogenic pulmonary edema. It is also the leading cause of death in COVID-19 patients [6,7]. A review of systematic analyses in 2008 showed an overall mortality rate of 43% for patients with ARDS of different causes across all the studies it included, and another meta-analysis from 2009 showed a mortality rate of 44.3% [8,9]. Current treatments for ARDS are limited and effective drug therapies are lacking [10,11,12]. Mechanical ventilation is often used to assist with breathing [13,14]. However, it has been argued that developing therapeutic strategies such as protective mechanical ventilation techniques may increase mortality in ARDS patients [15].

The pathogenesis of ARDS primarily involves a sustained inflammatory response, disruption of the alveolar epithelial and endothelial barriers, and extensive activation of neutrophils and platelets [16,17]. Neutrophil elastase has been identified as a key factor in causing vascular endothelial cell damage and increasing vascular permeability, playing an essential role in the development of ARDS [17,18]. Sivelestat sodium is a proven selective neutrophil elastase inhibitor and has been used in clinical settings, particularly for patients with ARDS or Acute Lung Injury (ALI) [19]. Some studies suggest that sivelestat sodium can reduce the infiltration and activation of inflammatory cells, effectively inhibit the production of inflammatory factors, reduce lung injury, prolong the survival time of ALI patients, and reduce mortality rates [20,21]. A recent study has demonstrated a reduction in mortality in ARDS patients through the use of sivelestat sodium, with a significant improvement in mortality in patients with oxygenation index (PaO_2_/FiO_2_) ratios above 200 mmHg [20]. However, the efficacy of sivelestat sodium in ARDS patients has been controversial.

This retrospective study aimed to analyze the effectiveness and prognosis of sivelestat sodium in patients with COVID-19-related ARDS.

## 2. Results

### 2.1. Baseline Data Analysis

A total of 110 adult inpatients (87 males and 23 females) with COVID-19 combined with ARDS were included in the study. In total, 45 patients were treated with sivelestat sodium (sivelestat group) and 65 patients were not (control group). The median duration of medication in the sivelestat group was 6 days, ranging from a minimum of 3 days to a maximum of 15 days. Overall, 76 out of 110 patients died (69.1%) during the course of the study, 28 patients in the sivelestat group (62.2%) and 48 patients in the control group (73.8%). However, this difference in mortality rates between the two groups was statistically non-significant. At admission, the median PaO_2_/FiO_2_ ratio was 157 mmHg in the sivelestat group and 202 mmHg in the control group (*p* < 0.05). Regarding laboratory indicators, the mean white blood cell (WBC) count at the time of admission was 11.8 × 10^9^/L in the sivelestat group and 8.39 × 10^9^/L in the control group (*p* < 0.05). There was no statistically significant difference in age, gender, length of hospitalization, smoking, alcohol consumption, treatments, comorbidities, and vital signs between patients in the sivelestat and control groups. (Table 1).

### 2.2. Effect of Sivelestat Sodium on Inflammatory Indices and Oxygenation Indices

After three and seven days of sivelestat sodium treatment, the levels of inflammatory markers, including C-reactive protein (CRP), interleukin-6 (IL-6), and procalcitonin (PCT), showed varying degrees of reduction compared to baseline levels. Specifically, after three days of treatment, CRP decreased by 7.5 mg/L in the sivelestat group and 5.9 mg/L in the control group. IL-6 decreased by 4.1 ng/L in the sivelestat group and 6.8 ng/L in the control group. PCT decreased by 0.65 ug/L in the sivelestat group and 0.14 ug/L in the control group. After seven days of treatment, CRP decreased by 7.6 mg/L in the sivelestat group and 6.1 mg/L in the control group. IL-6 decreased by 2.52 ng/L in the sivelestat group and 4.3 ng/L in the control group. PCT decreased by 0.39 ug/L in the sivelestat group and 0.68 ug/L in the control group. However, despite these reductions, there was no significant difference in the reduction in these inflammatory markers between the sivelestat group and the control group.

**Figure 1:**
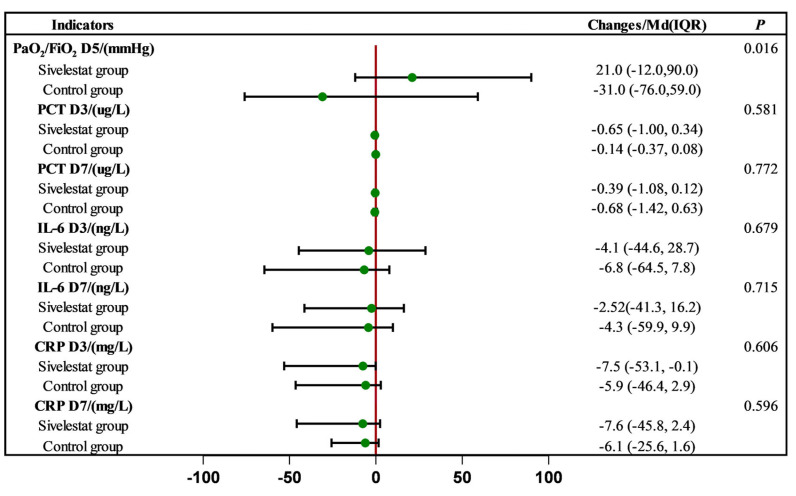
Effect of sivelestat on inflammatory indices and oxygenation indices.

In terms of oxygenation indices, after five days of treatment, the median oxygenation index in the sivelestat group increased by 21 mmHg, compared to the baseline values. In contrast, the control group experienced a decrease of 31 mmHg. This difference in oxygenation indices between the two groups was statistically significant (*p* = 0.016) (Figure 1).

### 2.3. Effect of Sivelestat Sodium on Clinical Endpoint Events

We investigated the effect of sivelestat sodium on inflammatory indices. It was found that, at admission, a considerable number of patients (100 for IL-6, 40 for PCT, and 87 for CRP) had levels higher than the normal range. After seven days of treatment, a portion of patients (16 for IL-6, 15 for PCT, and 19 for CRP) showed a reduction in their levels, which fell within the normal range. However, after adjusting for the relevant influencing factors, there was no significant difference observed between the sivelestat group and the control group regarding the chances of achieving normal levels of IL-6, PCT, and CRP on day 7. Similarly, this particular study did not find any statistically significant differences in terms of mortality between the sivelestat group and the control group.

**Figure 2:**
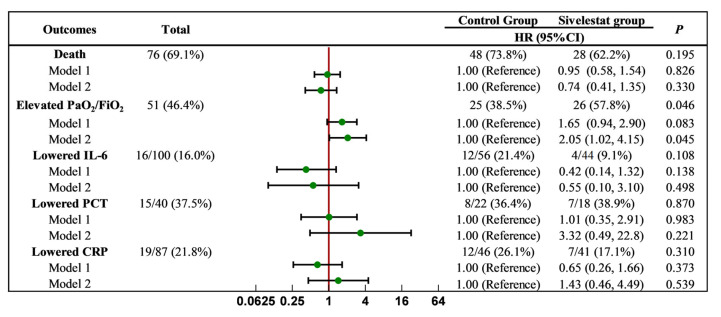
Effect of sivelestat on clinical endpoint events. Model 1, adjusted for age, gender, comorbidities, and smoke and alcohol status. Model 2, adjusted for all the factors in Model 1, and relevant examination indicators, including PaO_2_/FiO_2_, ALT, AST, Alb, Cr, BUN, WBC, IL-6, CRP, PCT, Hb, and PLT, except for the factor which was analyzed.

We also analyzed whether sivelestat sodium could improve the oxygenation index. The results revealed that a total of 51 patients (46.4%) showed improvement in their oxygenation index after treatment, with 26 patients (57.8%) in the sivelestat group experiencing an increase in this measure. This rate was significantly higher than the 38.5% observed in the control group (*p* < 0.05). Our findings demonstrated that the likelihood of an increase in the oxygenation index in the sivelestat group was 1.65 times higher than in the control group (Model 1, 95% CI = 0.94–2.90), although this difference did not reach statistical significance. However, after correcting for the relevant influencing factors, patients in the sivelestat group were 2.05 times more likely to exhibit an increase in the oxygenation index after five days of treatment compared to those in the control group. Furthermore, this difference was statistically significant (Model 2, 95% CI = 1.02–4.15, *p* < 0.05). (Figure 2).

Further analysis was conducted based on the patients’ baseline oxygenation index (PaO_2_/FiO_2_) levels. We divided the patients into two subgroups based on their oxygenation index.

Among the patients with a baseline oxygenation index of ≥ 200 mmHg, 23 out of 70 patients showed an increase in the oxygenation index (32.9%), while 48 patients died (70%). There was no significant difference observed in the rate of increase in the oxygenation index between the sivelestat group and the control group (Model 1, HR = 1.71, 95% CI = 0.73–3.97). However, after adjusting for relevant influencing factors, patients in the sivelestat group were 3.02 times more likely to experience an increase in the oxygenation index compared to those in the control group (Model 2, 95% CI = 0.92–9.94, *p* < 0.05). Additionally, there was no significant difference in terms of mortality between the two groups of patients (Model 1, HR = 1.22, 95% CI = 0.65–2.30; Model 2, HR = 0.51, 95% CI = 0.22–1.22).

**Figure 3:**
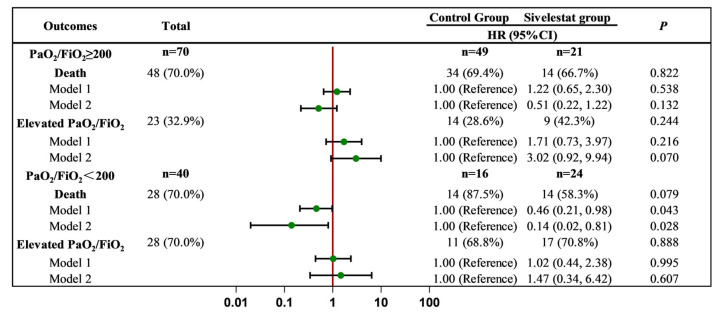
Subgroup analysis of the effect of sivelestat on clinical endpoint events. Model 1, adjusted for age, gender, comorbidities, and smoke and alcohol status. Model 2, adjusted for all the factors in Model 1, and relevant examination indicators, including PaO_2_/FiO_2_, ALT, AST, Alb, Cr, BUN, WBC, IL-6, CRP, PCT, Hb, and PLT, except for the factor which was analyzed.

Among the patients with a baseline oxygenation index <200 mmHg, 28 out of 40 patients showed an increase in the oxygenation index (70%), while 28 patients died (70%). Cox regression analysis revealed that the mortality rate in the sivelestat group was 0.46 times lower than that in the control group (Model 1, 95% CI = 0.21–0.98, *p* < 0.05). After adjusting for the relevant influencing factors, patients in the sivelestat group were 0.14 times more likely to die compared to those in the control group (Model 2, 95% CI = 0.02–0.81, *p* < 0.05). In this subgroup, there was no significant difference in terms of oxygenation index between the two groups of patients (Model 1, HR = 1.02, 95% CI = 0.44–2.38; Model 2, HR = 1.47, 95% CI = 0.34–6.42) (Figure 3).

## 3. Discussion

ARDS is a severe complication of COVID-19 [22,23], and patients with COVID-19 can rapidly progress to ARDS within a short period of time [24,25]. The incidence of ARDS in COVID-19 patients has been reported to be around 18–30% [26], mainly characterized by refractory hypoxemia due to an imbalance in the ratio of pulmonary ventilation to blood flow. The mortality rate among COVID-19 patients who develop ARDS is high, reaching about 81% [6]. Neutrophil elastase plays a crucial role in the development of ARDS, and sivelestat sodium, as a neutrophil elastase inhibitor, can effectively inhibit its activity. By inhibiting neutrophil aggregation, adhesion, and infiltration, [15] sivelestat sodium can reduce pulmonary hemorrhage, exudation, and attenuate pulmonary edema [27]. Sivelestat sodium was marketed in Japan in 2002 and is now widely used in clinical practice. Its use has also been approved for patients with COVID-19 infections. Previous studies have shown that sivelestat sodium can shorten the duration of mechanical ventilation and improve lung function in patients with ARDS [10]. In this study, the efficacy of sivelestat sodium in patients with COVID-19-associated ARDS was analyzed, along with its impact on patient prognosis.

Currently, it is observed that ARDS, respiratory failure, and severe outcomes such as death are predominantly seen in elderly patients affected by COVID-19. This trend may be attributed to the mutation of the virus, resulting in the prevalence of highly contagious but less virulent strains. Additionally, younger patients without underlying health conditions generally have milder disease presentations and better prognoses. In contrast, elderly patients with pre-existing health conditions are more likely to develop a severe disease and ARDS. The 110 patients with COVID-19 combined with ARDS included in this study were predominantly elderly and had a relatively high proportion of comorbidities such as hypertension, diabetes, and cardiovascular disease. As a result, they exhibited poorer outcomes, consistent with findings from other studies. According to the Berlin definition of ARDS, mild ARDS is defined as 200 mmHg < PaO_2_/FiO_2_ ≤ 300 mmHg, and moderate ARDS is defined as 100 mmHg < PaO_2_/FiO_2_ ≤ 200 mmHg. In this study, based on the baseline oxygenation index levels of the two groups, it was suggested that patients in the sivelestat group had a higher severity of ARDS at the time of hospital admission compared to the control group.

Assessing the effectiveness of the pharmacological treatments for ARDS can be challenging due to the multifactorial nature of the disease. The effect of sivelestat sodium on the risk of death in patients with ARDS is still a subject of debate, and different studies have reported conflicting findings. Some studies have suggested that treatment with sivelestat sodium may reduce the risk of death in patients with ALI/ARDS [20,21,28]. For example, a large-sample study involving 4276 patients showed that sivelestat sodium treatment within seven days of hospital admission may improve the prognosis of patients with ALI/ARDS [11]. However, other studies have concluded that sivelestat sodium does not significantly affect the risk of death in ARDS patients [10,15,19]. Instead, these studies have shown that sivelestat sodium can lead to a reduced length of hospital stay and improvements in inflammatory markers and oxygenation indices in patients. It is worth noting that an international multicenter study has even suggested that sivelestat sodium may increase the risk of death in patients [17].

Our study showed that among patients with baseline oxygenation indices below 200 mmHg, the mortality rate in the sivelestat group was lower compared to the control group. The use of sivelestat sodium significantly reduced the risk of death by 86% in this subgroup. However, among patients with mild ARDS, no significant difference in mortality rate was observed between the sivelestat sodium and control groups, indicating that sivelestat sodium did not have a significant effect on the risk of death in this subgroup. The overall higher mortality rate observed in the patients included in the study may be attributed to the advanced age and high prevalence of comorbidities in the patient population. It is suggested that the deaths of patients with ARDS due to COVID-19 are not solely attributed to respiratory failure caused by ARDS, but also influenced by factors such as advanced age, multiorgan dysfunction, and inflammatory response. Our findings indicate that sivelestat sodium reduces the risk of death in patients with moderate-to-severe ARDS, potentially by alleviating ARDS severity, multiorgan dysfunction, and inflammatory response through the inhibition of neutrophil elastase enzyme activity. In patients with mild ARDS, where deaths related to respiratory failure are less frequent, even improvements in the oxygenation index may not prevent mortality due to multiple organ failure and systemic inflammatory response. The development and outcomes of COVID-19 patients are indeed associated with early intervention. Early utilization of treatments such as Paxlovid, mechanical ventilation, and antibiotics may influence patient outcomes. However, in our study, we did not observe any statistically significant differences in treatment-related factors between the two groups.

Several studies have demonstrated the positive effect of sivelestat sodium on improving oxygenation in patients with ARDS/ALI [10,29,30]. For example, a study by Seigo et al. involving 110 patients with ALI showed that sivelestat sodium was particularly effective in patients with ALI whose baseline oxygenation index was below 140 mmHg [19]. In this study, we also found that sivelestat sodium positively improved the oxygenation index in patients with ARDS due to COVID-19. Firstly, after five days of sivelestat sodium treatment, the oxygenation index in the sivelestat group increased compared to the pre-dosing period, while a slight decrease was observed in the control group. Secondly, a higher percentage of patients in the sivelestat group showed an improvement in the oxygenation index compared to the control group. Thirdly, after adjusting for potential influencing factors, the inclusion of sivelestat sodium in the treatment regime more than doubled the likelihood of an increase in oxygenation indices. However, further stratification of patients based on their baseline oxygenation indices revealed that the improvement in oxygenation indices with sivelestat sodium was more prominent in patients with mild ARDS. In patients with moderate-to-severe ARDS, the use of sivelestat sodium did not significantly increase the likelihood of improvement in oxygenation indices. This finding may be attributed to several factors, including the severity of lung lesions and the presence of combined infections with other pathogens in patients with moderate-to-severe ARDS. Patients in this category often experience respiratory failure due to severe lung infections, which can rapidly respond to conventional antibiotics and hormone treatments, resulting in more significant rebounds in oxygenation indices. On the other hand, patients with mild ARDS may have less severe lung lesions primarily caused by novel coronavirus infections, making conventional treatments less effective. In such cases, sivelestat sodium may improve oxygenation indices by inhibiting neutrophil elastase, reducing pulmonary hemorrhage, and decreasing exudation. It is important to note that the limited sample size in our study may have influenced the statistical significance of the findings. Therefore, future studies with larger sample sizes are needed to further investigate the impact of sivelestat sodium on oxygenation indices in patients with ARDS.

The pro-inflammatory cytokine IL-6 has a crucial role in the progression of ARDS. Additionally, inflammatory markers such as CRP and PCT are indicators closely related to the prognosis of ARDS patients. A small-sample, randomized, double-blind clinical study showed that sivelestat sodium reduced IL-6 levels in patients [31]. A recent study in China confirmed this finding in patients with COVID-19 combined with ARDS [5]. In contrast, our results showed that after 3 and 7 days of treatment, IL-6 levels in both the sivelestat sodium and control groups were reduced compared to baseline levels, but there was no significant difference in the reduction or values between the two groups. Similarly, there was no significant difference in CRP and PCT levels between the sivelestat group and the control group after sivelestat sodium treatment. These results suggest that sivelestat sodium did not have a significant effect on reducing these inflammatory markers after seven days of combined treatment. We considered that the reason for the lack of statistical differences may be attributed to the high percentage (94.5%) of patients treated with glucocorticoids in both the treatment and control groups. Glucocorticoids primarily function to suppress inflammatory responses, which could potentially mask the effects of sivelestat sodium on IL-6 and other factors.

It is important to note that our study had limitations, including being a single-center study with a small sample size. Therefore, further large-scale, multicenter, randomized controlled clinical studies are needed to validate the findings and provide more robust evidence regarding the efficacy of sivelestat sodium in COVID-19-associated ARDS.

## 4. Methods

### 4.1. Patients

**Figure 4:**
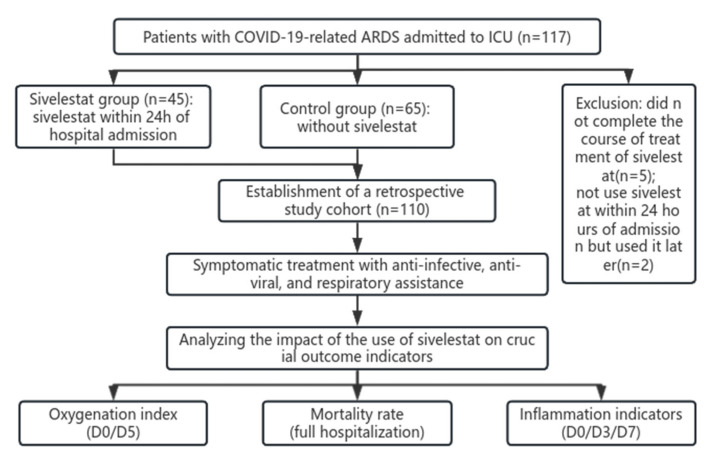
Enrollment process of COVID-19 patients with ARDS.

This study included 110 adult inpatients with COVID-19 who developed ARDS and were admitted to the ICU of the Fifth Medical Center of PLA General Hospital between 4 February and 15 July 2023. Signed written informed consent was obtained from all the participants. The principles of the Helsinki declaration were strictly adhered to during the implementation of this study, and the Committee on Human Research of the Chinese People’s Liberation Army General Hospital approved the study. The enrollment process of this study was shown in Figure 4.

The following inclusion criteria were applied:

1. All patients were diagnosed with severe or critical types of COVID-19 based on the Diagnostic and Treatment Protocol for COVID-19 (Trial 10th Edition).
2. All patients met the diagnostic criteria for ARDS as defined in Berlin definition.
3. All patients were 18 years of age or older.

The following exclusion criteria were applied:

1. Minor patients (below 18 years old).
2. Breastfeeding patients or pregnant women.
3. Patients who did not complete a full course of treatment with sivelestat sodium but refused to continue its use.
4. Patients who did not receive sivelestat sodium within 24 h of admission but used it in the middle and late stages of the disease.
5. Individuals who experienced adverse drug reactions to sivelestat sodium.

### 4.2. Diagnosis of COVID-19 and ARDS

The diagnosis of COVID-19 in these patients was based on the COVID-19 Diagnostic and Treatment Protocol (Trial 10th Edition) (1). Adults classified as severe cases meet any of the following criteria: shortness of breath with a respiratory rate (RR) of 30 times/minute or higher; resting oxygen saturation (SO_2_) of 93% or lower on air inhalation at rest; PaO_2_/FiO_2_ ratio of 300 mmHg or lower; and worsening clinical symptoms, with significant progression of lesions (>50%) within 24 h on imaging. (2) Adults classified as critical cases meet any of the following criteria: respiratory failure requiring mechanical ventilation and/or shock, combined with other organ failure necessitating ICU care.

The presence of ARDS in the patient was determined according to the Berlin definition of ARDS [32]. (1) Acute or progressive dyspnoea with a clear trigger within one week; (2) infiltrative shadows observed in both lungs on pulmonary radiograph/CT, which cannot be solely explained exclusively by pleural effusion, lobar/total lung atelectasis, or nodular shadows; and (3) hypoxemia, classified into three categories based on the PaO_2_/FiO_2_: mild, moderate, and severe. Mild: 200 mmHg < PaO_2_/FiO_2_ ≤ 300 mmHg; moderate: 100 mmHg < PaO_2_/FiO_2_ ≤ 200 mmHg; severe: PaO_2_/FiO_2_ ≤ 100 mmHg.

### 4.3. Grouping and Method

Patients were divided into two groups: the sivelestat group (n = 45) and the control group (n = 65), based on whether they received treatment with sivelestat sodium or not. Data on various parameters including gender, age, length of hospitalization, duration of sivelestat sodium use, outcome, vital signs, underlying diseases, treatments received, and laboratory test results were collected for each patient.

The main objective of this retrospective study was to compare the differences in clinical indices, test indices, and mortality rates between the sivelestat group and the control group. Additionally, the study aimed to analyze the effects of sivelestat sodium on patient outcomes, inflammatory indices, and oxygenation indices in individuals with ARDS associated with COVID-19. Cox regression analysis was used to perform these analyses.

### 4.4. Statistical Analysis

The software SPSS (version 26.0; IBM, Chicago, IL, USA) was used for data analysis. Frequencies and percentages (n, %) were used to express count data, and inter-sample comparisons were conducted using the chi-square test (x^2^). Measurement data using the Kolmogorov–Smirnov (K–S) test, and data that followed a normal distribution were expressed as mean ± standard deviation ($\bar{x}$ ± s), and comparisons between samples were made using the independent samples t-test. Data that did not following normal distribution were expressed as median with interquartile range (Md(IQR)), and comparisons between samples were made using the Mann–Whitney U test. Cox regression models were employed to evaluate the association between treatment with sivelestat sodium and the primary clinical outcomes.

## 5. Conclusions

In general, sivelestat sodium significantly reduces the risk of death in patients with severe COVID-19 infection-associated ARDS, who have a baseline oxygenation index of less than 200 mmHg. Sivelestat sodium also significantly improves the oxygenation index in patients with COVID-19 infection-associated ARDS, especially in patients with a baseline oxygenation index of not less than 200. However, sivelestat sodium does not have a role in the improvement of inflammatory markers in patients with severe COVID-19 infection-associated ARDS.

## Figures and Tables

**Table 1:** Characteristics of the study population according to the use of sivelestat.

	Total (n = 110)	Control Group (n = 65)	Sivelestat Group (n = 45)	*p* Value
Age/Md(IQR), (years)	84.0 (69.0, 91.0)	88.0 (71.5, 91.5)	77.0 (68.5, 88.0)	0.055
Male/(n,%)	87 (79.1%)	55 (84.6%)	32 (71.1%)	0.087
Length of hospitalization/Md(IQR), (days)	17.0 (10.0, 33.25)	19.0 (8.5, 35.5)	16.0 (13.0, 29.0)	0.910
Sivelestat-using days/Md(IQR), (days)			6.0 (5.0, 8.5)	
Smoking (n,%)	33 (30.0%)	20 (30.8%)	13 (28.9%)	0.832
Alcohol (n,%)	21 (19.1%)	12 (18.5%)	9 (20.0%)	0.840
Treatments (n,%)				
Mechanical ventilation	110 (100%)	65 (100%)	45 (100%)	
CRRT	36 (32.7%)	20 (30.8%)	16 (35.6%)	0.599
Antibiotics	109 (99.1%)	64 (98.5%)	45 (100.0%)	0.403
Glucocorticoid	104 (94.5%)	63 (96.6%)	41 (91.1%)	0.187
Immunoglobulin	19 (17.3%)	11 (16.9%)	8 (17.8%)	0.907
Vasopressor	78 (70.9%)	46 (70.8%)	32 (71.1%)	0.969
Paxlovid	57 (51.8%)	37 (56.9%)	20 (44.4%)	0.198
Comorbidities (n,%)				
Hypertension	68 (61.8%)	43 (66.2%)	25 (55.6%)	0.261
Diabetes	44 (40.0%)	30 (46.2%)	14 (31.1%)	0.113
Congestive heart failure	45 (40.9%)	30 (46.2%)	15 (33.3%)	0.179
Chronic pulmonary disease	17 (15.5%)	12 (18.5%)	5 (11.1%)	0.294
Chronic renal disease	17 (15.5%)	9 (13.8%)	8 (17.8%)	0.575
Liver disease	18 (16.4%)	9 (13.8%)	9 (20.0%)	0.391
Tumor	17 (15.5%)	13 (20.0%)	4 (8.9%)	0.113
Cerebrovascular disease	36 (32.7%)	22 (33.8%)	14 (31.1%)	0.764
Others	57 (51.8%)	33 (50.8%)	24 (53.3%)	0.791
Death (n,%)	76 (69.1%)	48 (73.8%)	28 (62.2%)	0.195
Vital sign ($\bar{x}$ ± s)				
Body temperature (°C)	36.9 ± 0.7	36.9 ± 0.7	37.0 ± 0.8	0.695
Heart rate (/min)	86.6 ± 16.0	88.0 ± 16.4	84.6 ± 17.8	0.311
Systolic blood pressure (mmHg)	133.4 ± 23.5	134.6 ± 21.6	131.8 ± 26.3	0.552
PaO_2_/FiO_2_ (mmHg) D0	178.0 (109.0, 261.0)	202.0 (112.0, 295.0)	157.0 (104.0, 213.0)	0.044
PaO_2_/FiO_2_ (mmHg) D5	206.0 (135.0, 280.0)	192.0 (129.0, 256.0)	211.0 (160.0, 272.0)	0.430
Laboratory indicators/Md(IQR), ($\bar{x}$ ± s)				
WBC(×10^9^/L)	9.79 ± 5.60	8.39 ± 4.06	11.80 ± 6.83	0.004
Hb(g/L)	121.4 ± 22.3	125.0 ± 21.7	118.9 ± 23.2	0.336
PLT/(×10^9^/L)	157.3 ± 39.4	161.7 ± 42.7	150.9 ± 36.0	0.484
Alb/(g/L)	30.2 ± 4.3	30.7 ± 4.0	29.3 ± 4.6	0.076
ALT/(U/L)	25.0 (17.0, 45.9)	23.1 (17.4, 44.2)	28.4 (16.1, 47.5)	0.308
AST/(U/L)	36.5 (24.8, 61.3)	35.2 (23.4, 49.1)	38.9 (26.6, 75.5)	0.315
Cr/(umol/L)	80.5 (59.8, 120.3)	82.8 (66.2, 121.5)	67.5 (58.9, 126.7)	0.335
BUN/(mmol/L)	8.87 (6.71, 15.30)	8.37 (6.79, 14.54)	9.22 (6.35, 16.13)	0.615
PT/(s)	14.6 ± 5.3	14.3 ± 2.8	15.2 ± 7.6	0.399
CRP/(mg/L)				
D0	34.7 (10.4, 91.5)	33.5 (10.3, 96.1)	35.1 (10.0, 90.4)	0.870
D3	19.3 (6.0, 50.2)	25.7 (7.7, 50.8)	15.1 (5.7, 50.3)	0.373
D7	18.5 (4.0, 56.3)	23.4 (5.1, 77.0)	16.3 (3.7, 51.5)	0.352
IL-6/(ng/L)				
D0	36.3 (21.1, 121.5)	33.7 (16.1, 135.3)	39.2 (20.3, 91.6)	0.51
D3	21.8 (13.2, 69.0)	20.5 (9.4, 72.7)	24.2 (12.7, 68.9)	0.332
D7	24.0 (16.9, 92.6)	22.2 (7.9, 76.4)	26.8 (17.2, 115.0)	0.236
PCT/(ug/L)				
D0	0.28 (0.07, 1.28)	0.27 (0.08, 1.00)	0.31 (0.04, 1.99)	0.447
D3	0.18 (0.05, 0.82)	0.18 (0.05, 0.69)	0.24 (0.03, 0.91)	0.329
D7	0.21 (0.06, 0.93)	0.12 (0.08, 0.84)	0.34 (0.03, 1.25)	0.336

Abbreviations: CRRT: continuous renal replacement therapy; WBC: white blood cell; Hb: hemoglobin; PLT: platelets; Alb: albumin; ALT: alanine aminotransferase; AST: aspartate transaminase; Cr: creatinine; BUN: blood urea nitrogen; PT: prothrombin time; CRP: C-reactive protein; IL-6: Interleukin-6; PCT: procalcitonin.

## Data Availability

Due to privacy concerns, we are unable to share the data. Please reach out to the corresponding author for any data inquiries.
